# SMS-Based Medical Diagnostic Telemetry Data Transmission Protocol for Medical Sensors

**DOI:** 10.3390/s110404231

**Published:** 2011-04-08

**Authors:** Ben Townsend, Jemal Abawajy, Tai-Hoon Kim

**Affiliations:** 1 School of Information Technology, Deakin University, Pigdons Road, Geelong, VIC 3217, Australia; E-Mail: bjt5150@gmail.com (B.T.); jemal@deakin.edu.au (J.A.); 2 Department of Multimedia Engineering, Hannam University, 133 Ojeong-dong, Daedeok-gu, Daejeon 306-791, Korea

**Keywords:** data transmission, data communications, SMS, short message service, GSM, mobile communications, information security, information privacy, telemetry, medical telemetry

## Abstract

People with special medical monitoring needs can, these days, be sent home and remotely monitored through the use of data logging medical sensors and a transmission base-station. While this can improve quality of life by allowing the patient to spend most of their time at home, most current technologies rely on hardwired landline technology or expensive mobile data transmissions to transmit data to a medical facility. The aim of this paper is to investigate and develop an approach to increase the freedom of a monitored patient and decrease costs by utilising mobile technologies and SMS messaging to transmit data from patient to medico. To this end, we evaluated the capabilities of SMS and propose a generic communications protocol which can work within the constraints of the SMS format, but provide the necessary redundancy and robustness to be used for the transmission of non-critical medical telemetry from data logging medical sensors.

## 1. Introduction

The ability to remotely monitor patient vital signs in real time is a growing area of interest [1]. Portable medical monitoring devices are used in hospitals with short range transmission infrastructure to allow patient sensors to communicate with base-stations. The typical use of such sensors sees them worn by the patient and paired with an aggregating transmitter using either fixed land line services or expensive mobile data transmissions. Sensors can be used in the home or other remote locations for the remote monitoring of non-critical care patients. While sensors and base stations allow a patient to be monitored externally to the hospital environment, it does not allow them to have any real degree of mobility in that the patient is still effectively tethered to the base station and the communications infrastructure. However, in some cases it should have been possible to monitor patients remotely, given the correct sensors and communications infrastructure to allow their status to be regularly checked.

The trend within patient monitoring has been to allow the patient more mobility [2]. Home monitoring is one of the possible medical applications of the mobility-aware healthcare systems [3–5]. For people to be able to stay in their home environment and feel secure and live as a normal life as possible is important for recovery. The contemporary and emerging developments in wireless communications and network technologies coupled with advances in ubiquitous and wearable technologies will have a radical impact on future health-care delivery systems. These advances have already made a significant impact on current e-health and telemedical systems. Numerous systems for elderly care [6], epilepsy care [7], and patient monitoring [8] based on mobile phones [9], Wi-Fi [8,10], and wireless sensor networks (WSN) [11] have been developed. Networked systems like these are of great economical benefit for the society as it would reduce health care costs.

A system to remotely monitor a patient’s blood pressure is described in [11]. The data is transferred to a central monitoring station using a wireless sensor network for display and storage. MEDiSN [12] and SMART [8] focus on monitoring patients waiting in emergency rooms. The network architectures in MEDiSIN are stationary relay nodes deployed to ensure connectivity between a patient worn sensor and a base station. AlarmNet [6] supports the collection of data from mobile and static sensors through a query service. Chipara *et al.* [13] discussed the design, deploymentand empirical study of a wireless clinical monitoring system that collects pulse and oxygen saturation readings from patients in general hospital units. It is shown that WBAN based on a low cost wireless sensor network technology could greatly benefit patient monitoring systems in residential environments [14].

In this paper, we develop a multi-tier real-time patient monitoring system architecture that integrates medical sensors, a sensor network, electronic patient records and the Short Message Service (SMS) [15] as a data transfer mechanism to allow remote monitoring of patient physiological status in the homecare conditions and to alert medical personnel when life-threatening events occur. SMS is a flexible and pervasive messaging technology which is almost universally accessible to anyone possessing a mobile telephone of second generation or later lineage. With the ability of SMS to encode data in a number of formats, and the capability to expand the user data segment through concatenated SMS, SMS offers excellent capacity for data transmission and it can be considered suitable for the transmission of discrete data in a variety of applications. In the case of critical data, where delivery failure is simply not an option, SMS is not the ideal choice of carrier medium due to the lack of delivery guarantees and standardised delivery receipts supported in the protocol. To address this problem, we extended SMS with an appropriate message validity period, failover options and redundancies so that SMS could be considered as a transmission medium for a wide variety of data. The proposed system organisation allows flexible design space for optimum trade-off (e.g., between processing power and storage capacity) as well as able to provide a wires-free environment so as not to limit movement and attract undesired attention during continuous monitoring. Based on the architecture, we propose a generic communications protocol to work inside an SMS message and provide the necessary redundancy and robustness for the transmission of non-critical medical telemetry. Such telemetry could then be transmitted from a mobile telephone or other cellular mobile engine, and this capability could be used to increase the freedom of non-critical patients requiring monitoring.

The rest of the paper is organised as follows. In Section 2, the background information is discussed. A brief background in SMS and telemetric data as well as various methods of collecting them is given. The design requirements for patient monitoring system are also discussed. The medical telemetry system architecture for physiological data collection is described in Section 3. Section 4 discusses a generic communications protocol to work inside an SMS message and provide the necessary redundancy and robustness for the transmission of non-critical medical telemetry. Such telemetry could then be transmitted from a mobile telephone or other cellular mobile engine, and this capability could be used to increase the freedom of non-critical patients requiring monitoring. The conclusion and future directions is given in Section 5.

## 2. Background

At its most basic level, telemetry encapsulates data and allows measurements remotely, and sends those measurements to a location where they are of most use. Medical sensors are available to take measurements of such biometric data such as Temperature, Heart rate, Blood pressure, Respiration (breathing) rate, Blood oxygen concentration and Blood sugar concentration. These sensors can all be used to take discrete readings which lend themselves to digital encoding. As a result, many of the sensors above could be encoded within an SMS. However, there are also more “detailed” types of medical sensors, such as the electrocardiogram (ECG), which plots an entire cardiac activity cycle in the heart. This more detailed type of sensor produces a vast amount of data, and sensors like ECG’s are typically used to produce continuous, analogue data.

When dealing with a human body, the ranges of data we expect to receive can be set with relative accuracy. Table 1 shows the ranges of data for a selection of vital signs in the human body. By defining the ranges of data, we can define the size of the data element we must use to record the data. For example, if dealing with data in the range 0–255, the value is recordable in a single octet. If the range is just one greater, 0–256, we require an extra bit, and depending on the data coding scheme we use, this may mean we will possibly require the use of an entire extra octet. The challenge is how to use these types of data, and varying sample rates, to encode useful information into the data space provided by SMS.

There are many different sorts of data which can make up a set of medical telemetry. However, not all this data is suitable for transmission in a mechanism such as SMS. An SMS message is a packetized collection of 140 octets (1,120 bits) of binary data ordered into an extremely well defined transport mechanism. In recent years, SMS has become an integral part of our daily lives and is now a virtually ubiquitous service offered by mobile carriers worldwide. Furthermore, modern SMS has introduced the multi-part or concatenated message, where a single SMS message can theoretically consist of up to 255 sequentially chained SMS packets [16]. Although theoretically possible to chain 256 messages together into a single concatenated SMS, the nature of the transmission mechanism for SMS messaging does not necessarily facilitate such large messages. Issues such as the fact that SMS may not be delivered (due to their internal validity period), and the fact that the GSM network does not guarantee that SMS are delivered to the recipient in the order in which they were sent [17] all impact on the maximum practical size of concatenated SMS messages. Thus, issues around delivery reliability, validity periods of messages, and the time-to-live for an SMS message, the delivery guarantees not made by the network, and the lack of formal delivery receipts must all be considered when examining potential applications for SMS. Furthermore, the security of information must be a concern in environments where personal or privileged data is being transferred. Therefore, for SMS to be used as the telemetry data transmission protocol, each of these issues needs to be examined, and an appropriate mitigation is implemented.

An important technical challenge is the apparent issues of multi-sensor medical data fusion, system optimization, real-time wireless transmission, and information security. A medical telemetry data set may consist of data from different sensors. However, we could consider a case where a number of patients are monitored at the same location. In this case, a single telemetry dataset might well contain discrete data from sensors on many different patients. To cater for such situations, it was our intention to create a data coding scheme to cater for the following scenarios: (i) Single patient, single sensor; (ii) Single patient, multiple sensors; (iii) Multiple patient, multiple sensors.

Another major issue with medical telemetry is the data transmission reliability and security [18]. When monitoring a patient, we must have a high level of trust in the quality of the data. Thus, it is very important that the system collects and transmits data reliably, and in a timely manner to the monitoring entity. Reliability in a medical system network can be defined in terms of delay profile, delay jitter and information loss rate whereas efficiency of a network can be defined as the ratio of information bits to the total transmitted bits [19]. Also, it is important to emphasise that, in the case of medical monitoring applications, simply wearing the device may disclose to the adversaries such as patient’s employer or insurer or acquaintances that the patient is suffering from a medical condition [18]. Consequently, the wearable monitoring device has to be as unobtrusive as possible, to preserve patient’s privacy.

## 3. Telemetry System Architecture for Physiological Data Collection

**Figure 1.**
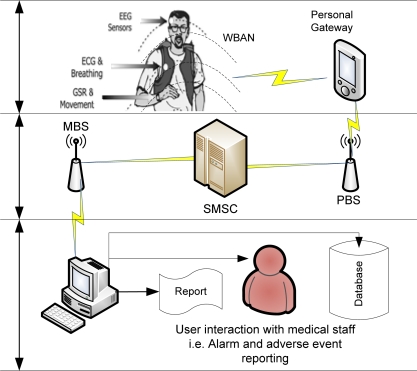
A medical telemetry system architecture for physiological data collection.

Mobility-aware healthcare systems are created as a synergy of emerging mobile computing, wireless medical sensors, and wireless communications and networks technologies. Figure 1 outlines the proposed telemetry transmission system where multiple physiological signals (such as ECG, EEG and GSR) are monitored using sensors and their state integrated using a low-power DSP based personal server. The whole system is integrated into hierarchically organised functional components. The medical centre base station (MBS) is a data aggregator and can be used to: Request updates from a patient, Receive regular (scheduled) updates from a patient, Report on alarms and adverse events and Store data.

The patient wears one or more wireless medical sensors with networking capability for physiological data collection. The sensor nodes measure and transmit patient vital sign information (e.g., the heart rate and blood oxygenation) to the local base station. Individual sensors would cover only limited range (in the order of ten feet) and therefore require very low power consumption for communication. Also, the sensors are so small that they will not affect the appearance and function of the patients in which they are embedded. Also, monitoring is carried out actively but unobtrusively without the patient or the people close by being aware of it. Moreover, the medical sensors make possible the combined processing of spatially and temporally gathered physiological information from different parts of the patient body and the external communication for mobile health care.

The medical sensors are connected to a GSM-compatible self-powered and wearable patient mobile personal gateway (PG) with the Internet access. The medical sensors will transmit data to PG which will subsequently transmit the combined data to a record server connected to a hospital network. PGs provide enough processing power for real-time data processing and analysis as well as reduced power consumption is achieved by employing mobile gateways. Individual sensors communicate with a PG using Bluetooth [12] or ZigBee [20]. The PG receives data from the sensors, logs this data to local memory, and then sends data using SMS over the network to a remote receiver. Standard SMS (and thus GSM network functionality) is used as the data carrier medium. Through the GSM network (or descendant networks such as 3G), the PG connects to the network, creates standard SMS message packets containing the encoded protocol, and sends data to the remote base station at the medical centre. Although WBAN based on a low cost wireless sensor network technology could greatly benefit patient monitoring systems in residential environments [14], the mesh networks do not require a fixed wired infrastructure, the cost of using a mesh network consisting of wireless sensors is lesser than that of using a Wi-Fi based system.

The system supports both scheduled transmission and polled requests from a remote base station at the medical centre. Alternatively, a message may be triggered by an adverse event which the base station is configured to look for. As it is impossible for a controller at hospital or relatives to make any sense or analyse a constant flow of patient data, the data should be forwarded to the hospital to be analysed only in critical cases, for example when the data diverge from the patient’s history. For example, a heart rate outside the normal adult range of 50–140 beats per minute may trigger an alarm message. The patient or doctor or both could formulate triggers that cause even more data to be collected, additional sensors to be enabled, or medical personnel to be contacted.

At the medical centre, the system will understand protocol messages and should be able to both read and write them. The medical centre base station may issue requests to remote patient stations and perform a number of operations with the data it receives. The base station should include capabilities to report on data being received, log the data to a database, and interact directly with a user (for example, a doctor) in the case of an adverse event, a medical emergency, or where a threshold condition is reached (such as heart rate exceeding a certain tolerance level).

It is important to note that, while the patient base station may send data regularly to the medical centre, the communication process is bi-directional. The medical centre may send a request to the patient’s local device, and the patient base station will respond to that request (a poll/response message pair). The protocol must therefore possess the capability of servicing a number of types of communication, including polls and responses, and scheduled communications. However, in addition to data messages, we should maintain the ability to control, configure and diagnose the remote system. The very concept of telemetry defines that we have remote devices measuring values. It may be necessary to turn on or off specific sensors, or change the frequency of data transmissions from the patient to the medical centre. We do not want to personally visit the patient each time such a change is required. With this in mind, Table 2 identifies the different message types.

The arrangement of patients to base stations is potentially a many to one relationship—that is, many patients should be able to be monitored from the same base station. Conversely, a single base station should have the ability to identify and receive transmissions from multiple patients simultaneously. As transmissions can be bi-directional (from medical centre to patient and vice versa), and there are many patients talking to a single base station, it is imperative that data are uniquely identifiable as to their point of origin and their purpose. That is, any datum must be associable with one, and only one, patient.

The proposed framework will be able to facilitate communication among patients, medical professionals at local hospitals and specialists available for consultation from distant places. The most valued features of this system is to prevent the patient from re-hospitalisation and to avoid possible critical events, thus reducing the healthcare costs. Another main advantage of the proposed framework is the unobtrusive patient vital sign monitoring. A pragmatic approach to achieving high end-to-end reliability is to isolate the impact of mobility from multi-hop routing. In our network architecture we divide the problem of data delivery from the sensor nodes to the base station into two parts: single-hop communication from the patient node to the first relay and (potentially multi-hop) communication from that relay to the base station.

Based on the architectural framework discussed in this section, we will propose a generic communications protocol to work inside an SMS message and provide the necessary redundancy and robustness for the transmission of medical telemetry. Such telemetry could then be transmitted from a mobile telephone or other cellular mobile engine, and this capability could be used to increase the freedom of non-critical patients requiring monitoring.

## 4. SMS-Based Telemetry Data Transmission Protocol

While the protocol defined in this section is ostensibly generic, the chosen application for our communications protocol is to send medical telemetry from a patient to the healthcare providers. For our protocol to have a purpose, there must be an appropriate implementation of a telemetry system and any such system should contain the following components integrated to address the following requirements:

- Allow the gathering of data from patient-based sensors.
- Provide a mechanism for the collection and formatting of the data into appropriate protocol messages.
- Allow for the transmission of the data to a remote monitoring site.
- Allow for the receipt and appropriate processing of the data at some remote point.

Moreover, we must consider overall capacity, the robustness and reliability of the medium, the availability of the carrier mechanism, and the tolerance of the system for faults and corruption. Ultimately, all of these factors must be considered in any system aiming to use SMS for the purposes of data transmission.

### 4.1. The Structure of the Protocol

**Figure 2.**
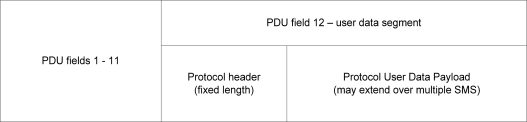
Fundamental structure of the SMS Protocol.

The SMS communications protocol fits within the confines of a single SMS message by using a structured header and an extensible user data payload. The user data payload may spread across the capacity of a concatenated SMS. The protocol is comprised of three main components (Figure 2).

The first element in the protocol is the SMS packet itself. Each SMS message is encapsulated in a packet called a PDU. The protocol is implemented in field 12 of the PDU, the user data segment. While we must be aware of the PDU’s structure and data, the PDU is pre-defined and is outside the scope of the protocol design, other than as a data source. Our protocol is not responsible for implementing the PDU packet, as this is done by the mobile device.

The second and third elements of the protocol can be considered to be the true protocol, and these sections are encoded in the PDU’s user data segment as the “SMS message” that we are sending. The second element is the protocol header. The protocol header contains consistent, structured information for any protocol message. This information is used for administration and control purposes (for example, identifying the message type, specifying the sender device ID, destination device ID, and so on). The header is a fixed length component of the protocol message, and is always located in the first SMS message of any protocol transmission (whether single SMS or concatenated SMS).

The third component of the protocol is the user data payload. This is a variable length field, containing application specific information based on the current use of the message. The data segment is used in an application specific way. For example, in medical telemetry, the user data segment may contain 100 heart rate samples for a patient, or an aggregated set of values from multiple sensors, or sensor readings for different patients all monitored at the same site.

### 4.2. Protocol Header

The header is entrusted with the management and “housekeeping” of the protocol. It is the responsibility of an application using the protocol to construct the header appropriately. The format of the header is strictly defined, and can be written to a fixed length data segment. This has advantages in that the application using the protocol can extract the header from the user data quickly, find the start and the end points of the header, and decompile the header into component fields without worrying about variable length field data in the header itself. In effect, the header can be extracted and “cookie cut” into components fields using the same mechanism for any implementation of the protocol.

The protocol header contains the following information:

- Synchronisation marker—start of protocol message
- Message Format and Protocol ID
- Message Type ID
- Message Structure—Multipart or Single Message
- Application ID—Protocol user information
- Sender Device ID—unique application ID of the sender
- Recipient Device ID—unique application ID of the recipient
- Message ID—sequential ID of this message
- Generation Timestamp—when was the message sent by the remote device
- Validity Period—how long should the message remain valid for
- Receipt Required Flags—is a receipt required, and if so, what type of receipt
- Control Octets—containing control messages for the remote device
- User Data Segment Length—how many octets of user data to follow
- User Data Segment Encryption ID—is the user data encrypted, and by what mechanism
- Header Checksum—checksum for header data only
- User Data Segment Checksum—checksum for user data only
- Message Checksum—checksum for entire message, including the other checksums

**Figure 3.**
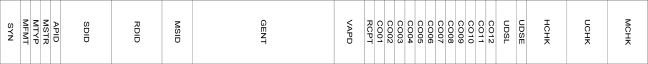
Field positions in the Protocol header.

The header is encoded in the order in which the fields are defined above. Figure 3 shows the location of each header field, relative to the start of the message:

The header requires 64 octets of the first message in a protocol message set. This leaves 76 octets of user data capacity in the first message.

### 4.3. User Data Segment

The user data segment is 76 octets in the first SMS message, as this message always contains the header. However, if we move into the realm of concatenated SMS, our protocol supports a concatenated SMS consisting of up to 6 SMS messages. In this case, all subsequent SMS after the first message provide their full payload for user data. With six SMS concatenated into a protocol message, we can access up to 740 octets of user data. The available user data payload can contain any sort of information, ultimately translated into 8 bit binary form. An application using the protocol may use 7 bit ASCII data, 8 bit binary or 16 bit Unicode. The exact format of the payload is specific to the application using the protocol.

In the case of critical data, where delivery failure is simply not an option, SMS is not the ideal choice of carrier medium due to the lack of delivery guarantees and standardized delivery receipts supported in the protocol. To address this problem, an appropriate message validity period, failover options (such as message receipting to validate delivery has occurred) and redundancies (such as application checks for receipts, fault and diagnostic messages) are proposed, with which SMS could be considered as a transmission medium for a wide variety of data. The architecture integrates medical sensors, a sensor network, electronic patient records and web portal technology to allow remote monitoring of patient physiological status in the homecare conditions and to alert medical personnel when life-threatening events occur. This way we are able to provide hassle-free environment for patients and other users, without excessive wires that limit movement and attract undesired attention during continuous monitoring. The proposed system organisation allows flexible design space for optimum trade-off between processing power, device power consumption and battery life, and storage capacity, sufficient for most medical applications. Based on the architecture, We proposes a generic communications protocol to work inside an SMS message and provide the necessary redundancy and robustness for the transmission of non-critical medical telemetry. Such telemetry could then be transmitted from a mobile telephone or other cellular mobile engine, and this capability could be used to increase the freedom of non-critical patients requiring monitoring.

### 4.4. Security

Another major issue with medical telemetry is that we are working with people’s lives and their ongoing wellbeing. When monitoring a patient, we must have a high level of trust in the quality of the data. To trust the data, we must ensure that what we transmit is what is received at the other end. Any data transmitted must be able to be validated at the destination to ensure that data integrity has been maintained. This implies the use of checksums to allow a recipient to check the content of a message and ensure it results in the same checksum value as is recorded in the message. Any difference between the checksum transmitted and the checksum calculated by the recipient implies an error in the transmission, and potential corruption in the data. A checksum must be used which will almost certainly result in an error being discovered as any transmission errors would require the data be discarded and retransmission requested.

The transmission of personal medial data implies certain privacy concerns. Obviously, most people are unlikely to want their medical data to be open to the public. A telemetry protocol should support encryption or obfuscation of data so as to prevent it being useful to anyone other than the intended recipient. While SMS is not highly secure, SMS messages can be considered secure from the casual eavesdropper due to the encryption which is natively present in SMS. However, the user data segment of an SMS can also be independently encrypted using a user defined method, so there is no reason why an SMS cannot be used for the transmission of personal and private information with user data encryption suited to the matter at hand. SMS has disadvantages, however. Issues around delivery reliability, validity periods of messages, and the time-to-live for an SMS message, the delivery guarantees not made by the network, and the lack of formal delivery receipts must all be considered when examining potential applications for SMS. Furthermore, the security of information must be a concern in environments where personal or privileged data is being transferred. Therefore, for SMS to be used as the telemetry data transmission protocol, each of these issues needs to be examined, and an appropriate mitigation is implemented. If this is accomplished, there is no real reason why SMS cannot be used for the transmission of important data. This protocol supports application specific encryption, and also provides a mechanism to use multiple different encryption schemes for the user data through the use of an encryption type octet.

### 4.5. Implementation

The protocol discussed in overview here was implemented as a theoretical application. While initial code has been generated to allow for protocol messages to be created, there is no real world implementation at this time. However, the next step in this research is to create a fully working, point to point implementation of the protocol, using real-world GSM modems and appropriately written software. This will include the creation of classes/objects to implement the protocol structure and encoding, and manage the transmission and reception of protocol messages of all different types. The ability for a user to encode and decode a message, and then read a message in human readable form when it is received will also be developed. An end-to-end implementation of the protocol could act as both trainer and simulator, allowing protocol users to develop application ideas and test them out in real world protocol messages, performing actual transmissions and checking all aspects of the data they wish to send.

The second development of this research will be the implementation of a system to make use of the protocol in a real world application. The system will be an implementation of a biometric data monitoring application with remotely monitored users transmitting data to a central base station. The various hardware devices for such a system exist right now, and for this research, we would undertake the definition of a new application for the component hardware, combining all of the disparate parts into a fully integrated working telemetry system using this protocol for its data transmission. Such a project would require the integration of medical sensor hardware with GSM compatible communications devices, and the implementation of remote and medical centre base station software and hardware to offer the appropriate levels of functionality to each type of user of the system. Finally, in terms of ongoing development, it is reasonable to believe that different versions of the protocol could evolve over time, to cater for specific needs and applications. The protocol discussed here, defined as version 1, is intended to be the foundation upon which a variety of applications can be built. This protocol is generic enough that it does not place unnecessary constraints on the application using it. However, it must be considered that, for some specific applications, variations to the protocol may be required to best address the business, control or data requirements of that application.

## 5. Conclusions and Future Directions

In this paper, we proposed a framework that integrates medical sensors, a sensor network, electronic patient records and web portal technology to allow remote monitoring of patient physiological status in the homecare conditions and to alert medical personnel when life-threatening events occur. Based on the architecture, we proposed an SMS-based medical diagnostic telemetry data transmission protocol for medical sensors. The standard SMS has several disadvantages such issues around delivery reliability, validity periods of messages, and the time-to-live for an SMS message, the delivery guarantees not made by the network, and the lack of formal delivery receipts must all be considered when examining potential applications for SMS. Furthermore, the security of information must be a concern in environments where personal or privileged data is being transferred. With the proposed extensions, it is possible to use SMS for the purposes of creating a formalised and highly structured communications protocol. This protocol, implemented within the confines of a single SMS message, but expandable into the realm of concatenated SMS messages for those messages which carry data, can be made sufficiently generic so as to be suitable for a wide variety of applications. We are currently studying the performance of a remote patient monitoring system using an OPNET based simulation model.

## Figures and Tables

**Table 1.** Vital signs data-summary of ranges and types of data.

**Type**	**Min**	**Max**	**Unit of measure**	**Data type**	**# Octets**	**Example**
Temperature	0	∼50	°C	Binary	1	00100101
Heart Rate	0	∼200	Beats per minute	Binary	1	00111100
Blood Pressure	0	∼200	mmHg (x 2 measurements)	Binary	2	01111000
						01010000
Respiration rate	0	∼50	Breaths per minute	Binary	1	00001110
Blood oxygen concentration	0	100	Percentage Oxygen	Binary	1	01100100
			Saturation (SpO_2_)			
Blood glucose concentration	0.0	∼50.0	Mmol/L–a decimal value (to 1 decimal place)	Binary coded ASCII	3	8.2

**Table 2.** Allowable values of the message type octet.

**Msg Type**	**Description**
1	Request message from base to remote device (poll)
2	Response message from remote device to base (poll response)
3	Remote originated message (*i.e.*, a message sent from the remote patient with no associated request from the medical centre)
4	Receipt message acknowledging receipt of an earlier message (using the message ID to identify the message that is being acknowledged)
5	Control message send
6	Control message receipt
7	Fault message
